# Ziclague^®^ (*Alpinia Zerumbet* oil) in patients with hereditary spastic paraplegia - the randomized controlled ZISPAST trial

**DOI:** 10.1186/s13023-025-04007-5

**Published:** 2025-09-24

**Authors:** Fabricio Diniz de Lima, Katiane Raisa Servelhere, Maria Fernanda Ribeiro Bittar, Carelis González-Salazar, Alberto Rolim Muro Martinez, Tatiana Benaglia, Benilton de Sá Carvalho, José Luiz Pedroso, Orlando Graziani Povoas Barsottini, Anamarli Nucci, Marcondes Cavalcante França

**Affiliations:** 1 Department of Neurology, University of Campinas (UNICAMP), Campinas, Brazil; 2 Department of Statistics, University of Campinas (UNICAMP), Campinas, Brazil; 3 Department of Neurology, Federal University of São Paulo (UNIFESP), Sao Paulo, Brazil

**Keywords:** Alpinia zerumbet, Hereditary spastic paraplegia, Spasticity, Gait, Clinical trial

## Abstract

**Background:**

Spasticity is a hallmark of hereditary spastic paraplegia (HSP) and contributes to gait impairment. *Alpinia zerumbet* oil (Ziclague^®^) is a topical anti-spastic agent approved in Brazil, but not yet explored in HSP. Then, it was designed a randomized, placebo-controlled, double-blind, crossover trial to evaluate the efficacy and safety of Ziclague^®^ in patients with HSP: the ZISPAST trial.

**Methods:**

Each participant was randomly assigned to receive 0.8 mL of Ziclague^®^ dermal applications (0.064 mL of *Alpinia Zerumbet* equally divided in each adductor magnus and each triceps surae) or placebo 0.9%. The primary endpoint was change from baseline in self-selected gait velocity and secondary endpoints included changes in maximal gait velocity, walking endurance, spasticity, muscle strength, Spastic Paraplegia Rating Scale, pain, fatigue, quality of life and post-treatment perceived change and general impression. Adverse events (AE) were also recorded.

**Results:**

Fifty-seven patients were enrolled, 37 (64.9%) of whom were men and 50 (87.7%) with pure phenotype. Mean age was 44 (± 11.6; range, 22 to 74), mean age of onset 23 (± 16.6; range, < 1 to 62) and mean disease duration 21 (± 13.1; range, 2 to 54) years. Compared to baseline, there were no significant between-group differences in primary and secondary outcomes. There were few AEs, all of them mild. Incidence of AE was similar between treatment arms (*p* = 0.56).

**Conclusions:**

Ziclague^®^ was safe in patients with HSP, but it was not able to improve gait velocity considering methods and protocol used.

**Trial registration number:**

U1111-1218-2539. Registered 28 August 2018, https://ensaiosclinicos.gov.br/rg/RBR-83xh37.

## Highlights

Positive studies led to a RCT of Ziclague^®^ in patients with HSP.

Dermal Ziclague^®^ did not improve motor, non-motor symptoms and quality of life.

Placebo effect occurred in cognitive domain of fatigue and maximal gait velocity.

Alternative outcomes might provide more benefits in trials to come in HSP.

0.8 mL of Ziclague^®^ dermal applications were safe and well-tolerated.

## Introduction

Hereditary spastic paraplegia (HSP) represents a large group of monogenic disorders with 93 causative genes identified to date [1–4]. The prevalence of this condition reaches up to 9.6 cases per 100,000 [5]. Much has been learned on the genetic underpinnings and mechanisms underlying HSP subtypes in the past decade, but unfortunately this has not yet translated into mechanistic or disease-modifying therapies for this burdensome disorder. Currently, symptomatic interventions seem closer to clinical application, such as anti-spastic agents. Lower limb spasticity is indeed the core feature in all HSP subtypes. It greatly contributes to gait dysfunction, postural abnormalities, pain and fatigue in individuals with HSP [6–8]. 

Even though there are many pharmacological and non-pharmacological agents to treat spasticity in general, there are insufficient studies specifically looking at HSP-related spasticity. Furthermore, the available reports rely upon small and heterogeneous cohorts, and variable endpoints [9, 10]. This is indeed the case of some reports using chemical denervation and neuromodulation [11–21]. So far, there are few randomized placebo-controlled trials involving subjects with HSP [22–24]. In one study, Diniz et al. found botulinum toxin to be safe, reducing the spasticity, but not effective in improving gait velocity and in the two others studies, neuromodulation also decreased spasticity, but did not change mobility and quality of life. So, management of HSP-related spasticity still represents an unmet medical need. Some recent and small studies have shown that the dermal application of the essential oil of *Alpinia zerumbet* (brand name: Ziclague^®^) improves spasticity in patients with cerebral palsy and stroke [25–31]. The active component within this medicine is a potent L-type calcium channel modulator [32, 33], with biological effects in both smooth and skeletal muscle. Ziclague^®^ has been approved as an adjunctive therapy for spasticity in 2014 by the Brazilian Health Regulatory Agency, its national agency of drug administration, under registration number 1,155,700,690,025, and since then, shown to be safe and convenient for clinical use according to the opinion of some clinicians and previous studies [25–31]. This medicine has not yet been tested in HSP, so we designed a randomized, placebo-controlled, double-blind, crossover trial to evaluate the clinical efficacy and safety of Ziclague^®^ in patients with HSP - the ZISPAST trial.

## Methods

### Patients selection and randomization

Patients were selected from the University of Campinas (UNICAMP) Neurogenetics Outpatient Clinic in Campinas, Sao Paulo, Brazil from September 2018 to November 2019, after institutional review board (IRB) approval and written consent. They needed to meet all of the following inclusion criteria: (1) clinical features of HSP: predominant or exclusive lower limb spasticity, with or without weakness and with pyramidal signs on neurological examination; (2) presence of resting and dynamic clinically-relevant spasticity in the following muscles: adductors and triceps surae with relatively preserved strength; (3) whole-exome sequencing (WES) revealing a likely pathogenic or pathogenic variant according to American College Medical Genetics in a known HSP-causing gene or inconclusive result (no variant found) with positive familial history in at least one more generation pedigree; (4) age between 18 and 80 years; (5) ability to walk for at least 14 m without stopping (assistive devices were permitted) and (6) exclusion of all acquired causes of spastic paraplegia (neural axis MRI, nerve conduction study and electromyography, cerebrospinal fluid analysis and infectious, inflammatory and metabolic screening were performed).

Patients could not meet any of the following exclusion criteria: (1) presence of signs and symptoms other than spasticity that significantly compromise the walking abilities, such as gait ataxia, lower motor neuron dysfunction, peripheral neuropathy or fixed muscle contractures; (2) to be wheelchair bound; (3) Modified Ashworth Scale (MAS) [34] greater than 3 in adductors, triceps surae, quadriceps or hamstrings; (4) Muscle strength assessed by the Medical Research Council (MRC) scale [35] less than 4 in adductors, triceps surae, quadriceps or hamstrings; (5) dementia and/or mental retardation; (6) current botulinum toxin injections or the last injection less than six months ago; (7) pregnancy or breastfeeding; (8) refusal to sign a consent form. Those using oral continuous medications were instructed to keep stable doses throughout the study.

All included patients were randomized using the simple randomization method with a 1:1 allocation ratio by a clinical-research nurse, on the website www.randomizer.org [36], to receive dermal applications of Ziclague^®^ or placebo. She was the only unblinded investigator and did not deal with patient care or outcome evaluation.

### Study design and intervention

ZISPAST was a pragmatic, randomized, placebo-controlled, double-blind, crossover, phase 3, investigator initiated trial performed at the UNICAMP Clinical Research Center. Ziclague^®^ (0.08 ml of *Alpinia zerumbet* essential oil/mL) is a antispasmodic medication in the pharmaceutical form of an oily solution, produced and manufactured in Brazil, stored in an aluminum bottle, for topical administration, via spray (0.2 ml of Ziclague^®^/spray) to the skin over the muscle of interest.

**Fig. 1:**
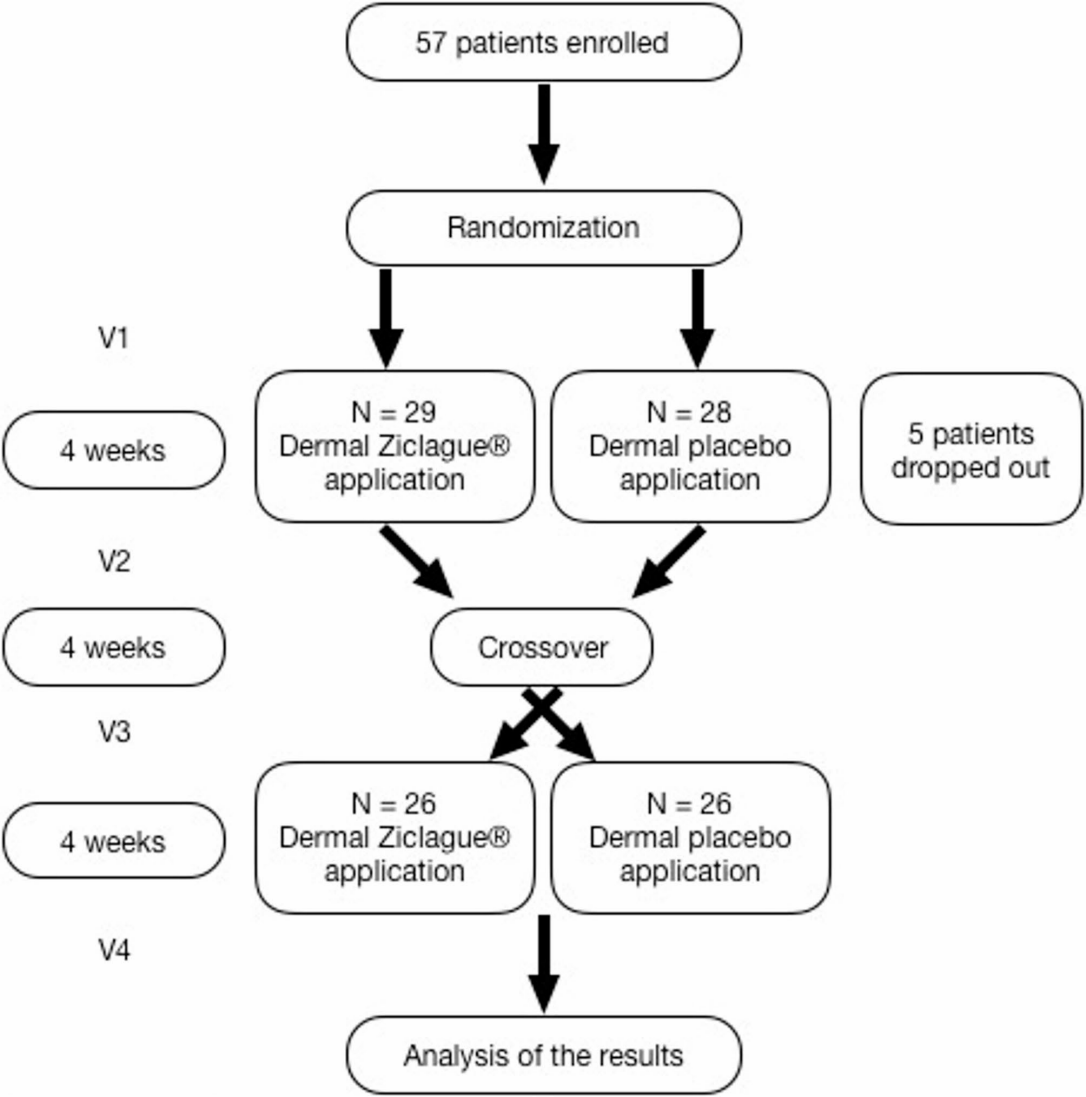
Study design and intervention. Left boxes represent the 4 visits (V1, V2, V3 and V4) in which patients were evaluated and the time intervals between evaluations

Enrolled participants were evaluated during 4 visits (Fig. 1): in the first (V1), they were evaluated for the outcome measures and were treated daily with either Ziclague^®^ (*Alpinia Zerumbet* essential oil) or placebo (vegetal oil vehicle without the drug) during 4 weeks. The measures were re-evaluated within 4 weeks in the second visit (V2) and a one-week interval was permitted. After 4 weeks, an arbitrary and safe time period to ensure total Ziclague^®^ washout, treatment groups were switched (crossover point). Then, in the third visit (V3), patients were evaluated once more and submitted to daily dermal applications thereafter for another 4 weeks. The final visit (V4) occurred 4 weeks after the second application course (Fig. 1). Considering the rarity of HSP and the comparison of the effects of treatment within subjects, a crossover design was chosen.

Each patient received four jet sprays of Ziclague^®^ (a total of 0.8 mL that corresponds to 0.064 mL of *Alpinia zerumbet* essential oil), one on each adductor and one on each triceps surae, or four jets of placebo (a vegetal oil vehicle without the drug and with physical aspects indistinguishable from its own), distributed in the same way. Medicine bottles were distributed by the nurse in a separate room according to the randomization and were delivered to the physiotherapist in order to perform the dermal application with her hands wearing gloves. During the procedure, she instructed the patient, clarified all their doubts so that she/he could perform the application alone at home and provided them with two boxes of 100 pairs of gloves. The same nurse made a daily telephone call to all patients to remind them to use the medication and ask them about the previous day, write down this information in a diary and ensure if they had any problems with administration and resolve any doubts, ensuring its daily use. The doses of two spray jets in each leg were used since these are the maximal doses on the registered dug label.

### Outcome measures

The primary outcome measure was change in gait at self-selected velocity 4 weeks after treatment. It was evaluated through the 10-meter walking test (10mWT), in which patients walked barefoot conforming with their comfortable velocity, used in everyday life, for a 14-meter distance (2 m accelerating, the middle 10-meter used for the measurement and 2 meters’ deceleration distance) [37], three times in a row and they were permitted to rest between each trial if needed. The self-selected gait velocity in meters per second was calculated based on mean time obtained from the three attempts.

Secondary outcomes included changes at 4 weeks’ post-treatment in the following: (1) maximal gait velocity with the same 10mWT protocol performed before; (2) walking endurance using 6-minute walking test (6MWT), in which patients walked twice and barefoot at their routinely self-selected gait, during 6 min in a 30 m stretch of unimpeded walkway, 60 min apart each one, and the mean distance was calculated. Total distance in meters was measured. A standardized encouragement was performed during the test, without influencing the patient’s walking velocity, and stops were permitted; (3) adductors as well as triceps surae were evaluated relative to changes in muscle tone (MAS, range 0–4) and strength (MRC, range 0–5); [34, 35] (4) Spastic Paraplegia Rating Scale (SPRS, range 0–52); [38] (5) monthly frequency of cramps and spasms; (6) Brief Pain Inventory (BPI), which was separated in two domains: pain severity (BPI-S, range 0–10) and pain interference (BPI-I, range 0–10); [39] (7) fatigue according to the total Modified Fatigue Impact Scale (MFIS, range 0–84) and also by calculating its individual subscale scores for physical (MFIS-P, range 0–36), cognitive (MFIS-C, range 0–40) and psychosocial (MFIS-PS, range 0–8) functioning; [40] (8) health-related quality of life, measured by the Short Form Health Survey (SF-36, range 0-100) [41], (9) post-treatment perception of change, evaluated by the Perceived Change Scale – Patient (PCS-P) [42], and (10) general impression of the treatment, in which patients were interviewed regarding general sensation of worsening, maintenance or improvement. The outcomes were evaluated by a single board-certified neurologist (gait velocity, strength, spasticity and SPRS) and a single neurology-trained physiotherapist (pain, fatigue, monthly frequency of cramps and spasms, quality of life, perception of change and general impression). Both were blinded and not involved in any other study-related procedures.

The team extensively emphasized to patients to regularly carry on their standard rehabilitation regimen, consisting of individualized physiotherapy program 3 times a week and additionally to perform the HSP-UNICAMP protocol of 3 sets of 45 s followed by a 15-second interval of balance (static and dynamic) and stretching (adductors, triceps surae, quadriceps, hamstrings and gluteus) exercises daily during all the course of the study, both as previously prescribed at UNICAMP outpatient clinic during their follow up. This protocol complements physiotherapy in order to maintain or improve muscle activation and mobility, optimize performance in routine activities and prevent muscle contractures and its consequences. Patients were trained by the physiotherapist to execute the sessions and received an illustrative folder before study initiation.

For safety assessment, patients were actively asked by the physiotherapist using a questionnaire regarding the occurrence of blood pressure reduction, somnolence, weakness, worsening gait, worsening falls, pain, local allergy and others. They received a diary to record the occurrence of adverse effects, cramps and spasms, which was previously instructed on how to use it to facilitate the reliability of the data and the interview.

### Statistical analysis

#### Sample size calculation

The necessary sample size calculation was based on previous study [14, 22]. They assumed a between-treatment effect of 12%, a power of 80% and a significance level at 0.05. Then, the estimated sample size was 49 patients. Considering a loss of follow up of 10% during the course of the study, the target sample size was at least 54 patients.

#### Efficacy and safety evaluation

Kolmogorov-Smirnov test was used to assess the data distribution. Succeeding, baseline characteristics and outcomes 4 weeks after each treatment were described after calculating means, standard deviations and range. Generalized estimating equations (GEE) were applied to compare the effect of the treatment with Ziclague^®^ versus placebo on the primary and secondary outcomes. Binary outcome (binomial distribution) was assumed for MAS, MRC and perception of change, which were measured on an ordinal scale, and a discretization indicating any worsening post-treatment was used. McNemar’s test with continuity correction was applied to compare adverse effects. For all the tests, it was assumed a significance level of α = 0.05. R software was used to analyze all data, according to the intention to treat principle, and Geepack library [43] was applied to fit GEE models.

## Results

### Baseline characteristics and randomization

Baseline characteristics were shown in Table 1. All the continuous variables presented a Gaussian distribution. The 10 patients with no variants found on WES, presented clinical features of HSP and positive familial history, according to the inclusion criteria.

**Table 1:** Baseline characteristics of patients

	Patients (*n* = 57)
**General features**	
Age, yo – mean value (SD; range)	44 (± 11.6; 22–74)
Age at onset, yo – mean value (SD; range)	23 (± 16.6; < 1–62)
Disease duration, years	21 (± 13.1; 2–54)
Sex, Men – number (%)	37 (64.9%)
Use of assistive device – number (%) **Phenotype**	49 (86.0%)
Pure, number (%)	50 (87.7%)
Complicated, number (%)	7 (12.3%)
**Type**	
* SPG4*	19 (33.3%)
* SPG3A*	6 (10.5%)
* SPG8*	5 (8.8%)
* SPG7*	3 (5.3%)
* X-linked adrenomyeloneuropathy*	2 (3.5%)
* SPG5*	2 (3.5%)
* SPG33*	2 (3.5%)
* SPG72*	2 (3.5%)
* SPG76*	2 (3.5%)
* SPG6*	1 (1.8%)
* PLA2G6-associated*	1 (1.8%)
* Inconclusive WES*	10 (17.5%)

Baseline characteristics of patients: general features, phenotype and type of HSP. SD, standard deviation; WES, whole exome sequencing; yo, years old

Five patients dropped out of the study (all before the second visit) because they were unable to attend the four visits. All patients using oral continuous medication kept stable doses throughout the study, as well as completing the rehabilitation program in accordance with what was instructed.

### Outcomes

**Fig. 2:**
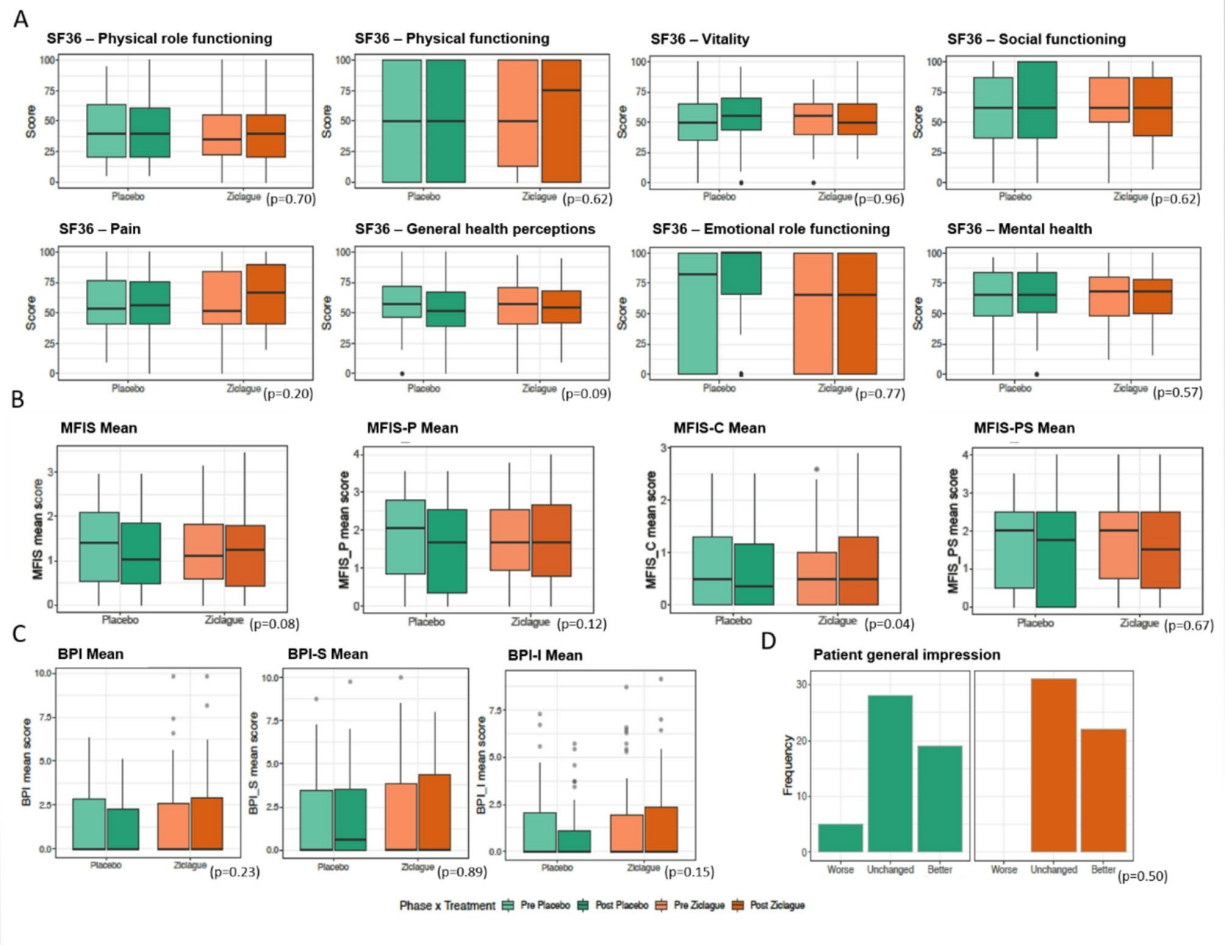
Non-motor secondary outcomes: quality of life, fatigue, pain and general impression of patients. Boxplots of (**A**) Short Form Health Survey domains, (**B**) Modified Impact Fatigue scale and its subscale scores, (**C**) Brief Pain Inventory and its domains and (**D**) patient general impression

The primary outcome, self-selected gait velocity, did not change after treatment with Ziclague^®^ when compared to changes observed after placebo dermal application (*p* = 0.17). Comparing maximal gait velocity, we could observe an increase in placebo group (*p* = 0.04). Changes did not occur in the other motor secondary outcomes, as show in Table 2. Regarding the non-motor secondary outcomes, we noticed lower MFIS-C scores in the placebo group compared to treatment (*p* = 0.04) and there were no statistically significant results in the other parameters, as illustrated in Fig. 2.

**Table 2:** Primary and motor secondary outcomes

	Ziclague^®^		Placebo		95% CI	SE	*p*-values
	Pre	Post	Pre	Post			
**Outcomes**	*N* = 52	*N* = 52	*N* = 52	*N* = 52			
**Primary outcome** ^**•**^							
Self-selected gait velocity (m/s)	0.85 (±0.31)	0.86 (±0.34)	0.81 (±0.31)	0.86 (±0.31)	[-0.07, 0.01]	0.02	0.17
**Secondary outcomes** ^**•**^							
Maximal gait velocity (m/s)	1.17 (±0.44)	1.17 (±0.45)	1.11 (±0.46)	1.17 (±0.46)	[-0.09, -0.01]	0.02	*0.04*
Endurance walking by 6MWT (m)	250.0 (±94.6)	249.3 (±103.4)	244.6 (±96.3)	249.6 (±95.1)	[-14.70, 4.90]	5.0	0.32
Spasticity (MAS)							
Hip adductors muscles	1.69 (±0.79)	1.54 (±0.68)	1.74 (±0.85)	1.61 (±0.72)	[-0.58, 1.02]	0.41	0.59
Triceps surae muscles	1.88 (±0.77)	1.86 (±0.79)	1.85 (±0.87)	1.73 (±0.82)	[-0.56, 1.04]	0.41	0.56
Muscle strength (MRC)							
Hip adductors muscles	4.60 (±0.66)	4.58 (±0.79)	4.50 (±0.82)	4.58 (±0.70)	NA	1.20	0.41
Triceps surae muscles	4.48 (±0.93)	4.54 (±0.79)	4.50 (±0.82)	4.56 (±0.82)	[-0.51, 2.19]	0.69	0.23

Primary and motor secondary outcomes: mean values (±standard deviation), 95% CI, standard error and p-values, obtained by GEE models. Considered on patients who completed at least two visits of the clinical trial (*N* = 52). 6MWT, 6-minute walking test; CI, confidence interval; MAS, modified Ashworth scale; MRC, Medical Research Council scale; NA, not applicable; SE, standard error

### Safety

Adverse events (AE) occurred in 18 patients after treatment with Ziclague^®^, involving blood pressure reduction, somnolence, weakness, worsening gait, worsening falls, pain, local allergy and others (burning, pricking or itching at the application site and nasal irritation or dizziness after inhalation). Despite this, all patients who participated in the 4 study visits were able to regularly administer the medication throughout the study period. Likewise, 22 patients experienced AE after placebo, 7 of which in both treatments (Table 3). The AEs were transient and tolerable, and the overall incidence was not significantly different between treatments (*p* = 0.56).

**Table 3:** Adverse events reported in ZISPAST trial

	Ziclague^®^	Placebo	*p*-value^•^
	*N* = 52	*N* = 52	
**Adverse events** ^**•**^	18 (35.3%)	22 (43.1%)	0.55
Blood pressure reduction	5 (9.8%)	4 (7.8%)	1.00
Somnolence	7 (13.7%)	8 (15.7%)	1.00
Weakness	1 (2.0%)	6 (11.8%)	0.13
Worsening gait	4 (7.8%)	4 (7.8%)	0.72
Worsening falls	1 (2.0%)	1 (2.0%)	0.48
Pain	2 (3.9%)	2 (3.9%)	0.62
Local allergy	2 (3.9%)	0 (0%)	0.48
Others	5 (9.8%)	6 (11.8%)	1.00

Number of patients who experienced adverse events (%). Considered on patients who completed at least two visits of the clinical trial (*N* = 52)

## Discussion

The ZISPAST trial set out to evaluate the efficacy and safety of Ziclague^®^ in patients with HSP, a rare group of neurodegenerative and progressive diseases of the pyramidal tracts with motor and non-motor manifestations [44]. Ziclague^®^ is an herbal drug with antispastic properties, administered topically by dermal applications, produced in Brazil and marketed since mid-2014. Its active ingredient is the *Alpinia zerumbet* essential oil, that is rich in terpenes, natural substances consisting of at least two compounds with the formula C_5_H_8_, which block muscle contraction in two ways: (1) by potent modulation of voltage-dependent L-type calcium channels, which are located in the transverse tubules, inhibiting the release of Ca + + from the sarcoplasmic reticulum through type 1 ryanodine receptors and (2) by promoting postsynaptic competition of acetylcholine in smooth muscles receptors, hence reducing muscle contraction and enabling synergism with other drugs [24, 45, 46]. There are some studies in the literature showing beneficial effects of Ziclague^®^ in reducing spasticity due to pyramidal lesions in patients with cerebrovascular accidents, cerebral palsy and spinal cord trauma [24, 47]. The ZISPAST trial, however, is the first study with Ziclague ^®^ specifically looking at lower limb spasticity and its impact on gait abilities.

Safety and outcomes were assessed based on a clinical rationale of analyzing the effect of treatment in (1) the main muscle groups involved in patients with HSP [48], which plays fundamental role in kinematics, velocity, width and walking quality [13, 16, 22, 49], (2) the main non-motor symptoms reported by patients, such as pain and fatigue [15, 50] and (3) quality of life and general perceptions. Accordingly, it was performed in a randomized, placebo-controlled, double-blind, crossover trial. In the design of this trial, the positive points and some tips from previous studies, especially the SPASTOX trial [22], were incorporated with the aim of minimizing limitations, increasing the reliability of the effects of Ziclague^®^ in these patients and making our approach as effective as possible. Even taking all these precautions into account, ZISPAST trial was a negative clinical trial. With the current dosing and administration protocol, it was not possible to achieve muscle relaxation and minimize its motor consequences nor to reach benefits on non-motor-symptoms. Despite that, the major lessons learned after this trial are: Ziclague^®^ is safe for patients with HSP and is also easy-to-use and adhere to. Likewise, running placebo-controlled clinical trials in HSP is something feasible.

The limitations of this trial enclose (1) the choice of muscles, that could be personalized through a biomechanical assessment [18], (2) the dose of Ziclague^®^, which despite being in accordance with the drug label and prior studies [24, 47], it may have been insufficient, given the possibility of differences in the body surface and muscle volume of the patients’ lower limbs, all of them influenced by variability of their weight and height [51–53], as well as age, previous muscular background [54, 55] and degree of amyotrophy [4] and (3) the lack of previous structured controlled trials with Ziclague^®^ to base the time needed for treatment to achieve its effects, considering studies with evaluations after a single dose, 4 weeks, 12 weeks and 8 to 28 weeks [25, 27, 30, 31]. Although the outcomes were negative, the ZISPAST trial was able to offer the following insights: (1) based on the scores of the applied scales and patients’ reports during the study period, we may verify that the frequency of non-motor complaints are really very common in patients with HSP, in spite of being often neglected [16, 50] and perhaps for this reason, proper attention is not given in clinical practice (and clinical trials) although they are important and necessary outcome measures, and (2) taking into account these results and those from all the previous randomized controlled trials [22, 43, 44], it seems that either the chosen outcomes are not the best for HSP or the spasmolytic effects achieved have statistical significance but without clinical repercussions or even both. In view of this, new therapeutic strategies are necessary and alternative outcomes, such as those obtained in a mobility lab using wearable sensors and even from a future reformulation of the SPRS scale could add more robust information, applicability and, consequently, provide more benefits in the design of trials to come.

In this scenario of negative outcomes, a placebo effect appears to have been observed particularly in the cognitive domain of fatigue (MFIS-C) and maximal gait velocity. The expressive majority of those subjects who presented improvement was in the first period of the study, i.e., the first two visits, when study participation was an exciting novelty. These results are in line with studies on processes and brain circuitry underlying placebo effects over emotional and motivational mechanisms that may induce a modulation of neurotransmitters, which in turn is capable of reducing anxiety and fatigue, thus improving attention, planning and organizational skills, such as learning [56]. These neurobehavioral issues, may have provided gains in motor performance, which translated during the trial into improvement in maximum gait velocity. It points out an indirect advantage of keeping up efforts to provide safe treatments available to spasticity in these patients, as well as researching new options.

## Conclusion

The ZISPAST trial found that the treatment of spasticity in patients with HSP using the drug Ziclague^®^ was not capable of reducing spasticity and producing functional improvement considering the application protocol and measuring instruments applied, despite a safe profile.

## Data Availability

The data that support the findings of this study are not openly available due to an ethical issue by the UNICAMP IRB. If there is interest, the corresponding author and chief investigator Prof. Dr. França Jr should be contacted to request the possibility of sharing with the UNICAMP IRB. Then, all individual de-identified participant data from the ZISPAST trial, its clinical protocol and statistical analysis plan will be shared with qualified researchers. Qualified researchers include those who agree to use the shared study data and materials ethically and exclusively for similar research or that contributes to the field of hereditary spastic paraplegia, the results of which will be made public promptly upon their generation.

## Abbreviations

AE: Adverse events
BPI: Brief pain inventory
BPI-I: Brief pain inventory, pain interference domain
BPI-S: Brief pain inventory, pain severity domain
GEE: Generalized estimating equations
HSP: Hereditary spastic paraplegia
IRB: Institutional Review Board
MAS: Modified Ashworth scale
MFIS: Modified fatigue impact scale
MFIS-C: Modified fatigue impact scale, cognitive subscale
MFIS-P: Modified fatigue impact scale, physical subscale
MFIS-PS: Modified fatigue impact scale, psychosocial subscale
MRC: Medical Research Council
NA: Not applicable
PCS-P: Perceived change scale - patient
SF-36: Short form health survey
SPG: Spastic Paraplegia
SPRS: Spastic paraplegia rating scale
UNICAMP: University of Campinas
WES: Whole-exome sequencing
10mWT: 10-meter walking test
6MWT: 6-minute walking test
